# RNA Binding Protein Motif 3 Inhibits Oxygen-Glucose Deprivation/Reoxygenation-Induced Apoptosis Through Promoting Stress Granules Formation in PC12 Cells and Rat Primary Cortical Neurons

**DOI:** 10.3389/fncel.2020.559384

**Published:** 2020-09-02

**Authors:** Wenwen Si, Zhen Li, Zifeng Huang, Shanyu Ye, Xinrong Li, Yi Li, Weihong Kuang, Dongfeng Chen, Meiling Zhu

**Affiliations:** ^1^Shenzhen Bao’an Traditional Chinese Medicine Hospital (Group), Guangzhou University of Chinese Medicine, Shenzhen, China; ^2^Department of Anatomy, The Research Center of Basic Integrative Medicine, Guangzhou University of Chinese Medicine, Guangzhou, China; ^3^Shenzhen Hospital of Integrated Traditional Chinese and Western Medicine, Guangzhou University of Chinese Medicine, Shenzhen, China; ^4^The First Clinical Medical College, Guangzhou University of Chinese Medicine, Guangzhou, China; ^5^Guangdong Key Laboratory for Research and Development of Natural Drugs, Key Laboratory of Research and Development of New Medical Materials of Guangdong Medical University, School of Pharmacy, Guangdong Medical University, Dongguan, China

**Keywords:** RBM3, G3BP1, stress granules, apoptosis, OGD/R

## Abstract

As a sensitive cold-shock protein, RNA binding protein motif 3 (RBM3) exhibits a neuroprotective function in the condition of brain injury. However, how RBM3 is involved in acute ischemic stroke by affecting stress granules (SGs) remains unclear. Here, we established an oxygen-glucose deprivation/reperfusion (OGD/R) model in rat primary cortical neurons and PC12 cells to explore the potential mechanism between RBM3 and SG formation in acute ischemic/reperfusion (I/R) condition. The immunofluorescence results showed that the SG formation significantly decreased in rat primary cortical neurons and PC12 cells during the reperfusion period after 6 h of OGD stimulation. The western blot results, flow cytometry analysis, and cell viability assessment showed that the RBM3 expression and ratio of cell viability significantly decreased, while the rate of apoptosis increased in PC12 cells during the reperfusion period after 6 h of OGD stimulation. Co-immunoprecipitation (Co-IP) and immunofluorescence indicated that RBM3 and GTPase-activating protein-binding protein 1 (G3BP1) colocalized cytoplasm of PC12 cells after 6 h of OGD stimulation when the SGs formation reached the highest level. Besides, overexpression and knockdown of the RBM3 were achieved via plasmid transfection and CRISPR-Cas9 technology, respectively. The results of overexpression and knockdown of RBM3 gene illustrated the pivotal role of RBM3 in affecting SG formation and apoptosis level in OGD-treated PC12 cells. In conclusion, RBM3 could combine with G3BP1 resulted in increasing stress granules generation in rat primary cortical neurons and PC12 cells after 6 h of oxygen-glucose deprivation (OGD) injury, which ultimately reduced the apoptosis in OGD-induced cells. Our study may enable a new promising target for alleviating ischemia-reperfusion injury in cells.

## Introduction

Acute ischemic stroke (AIS) is one of the most severe diseases, a leading cause of death and disability worldwide (Huber et al., 2019; Rossi et al., 2019; Wu et al., 2019). AIS triggers a damaging cascade as a multifactorial disease, including endoplasmic reticulum (ER) stress, oxidative stresses, excitotoxicity, inflammation, and apoptosis (Bettencourt and Ferro, 2020; Sui and Gao, 2020). Notably, ischemic cascade conditions exhibit very similar characteristics to stress granules (SGs) formation (Si et al., 2019). Stress granules are stalled translational aggregates generated in the cytoplasm and induced by varied environmental stress, including heat shock, hypoxia, ER stress, and oxidative stress (An et al., 2019; Guzikowski et al., 2019; Ivanov et al., 2019). The biological function of SGs formation is linked to protecting cells from cellular apoptosis, which could reduce the ischemia injury in AIS (Takahashi et al., 2013; Bittencourt et al., 2019). However, there have been very few studies on the function investigations of SGs related to AIS.

RNA binding proteins (RBPs) are essential molecules that could interact with both mRNAs and proteins and regulate the production of SGs (De Leeuw et al., 2007; Glisovic et al., 2008; Adeli, 2011). RNA binding motif protein 3 (RBM3) is one of the first discovered cold shock protein and belongs to RBMs’ family. Based on previous studies, RBM3 is an RNA-binding protein that associates with the various types of mRNAs, microRNAs, and RBPs (RNA binding protein) through the AU-rich element (ARE) in the 3′-UTR, which has a neuroprotective function in the condition of brain injury (Lunde et al., 2007; Hogan et al., 2008; Jackson et al., 2015; Zhu et al., 2016; Xia et al., 2018). Previous studies have shown that RBM3 knockdown can eliminate the neuroprotective effect induced by deep freezing, while RBM3 overexpression prevents cell death in serum starvation (Chip et al., 2011; Tong et al., 2013; Peretti et al., 2015). Besides that, RBM3 exerts an anti-apoptotic effect in regulating apoptosis through multiple signaling pathways, such as NF-κB, p38, PI3K/Akt, and JNK pathways (Yang et al., 2017; Zhuang et al., 2017; Ma et al., 2018; Ushio and Eto, 2018) and appears in SGs under stress conditions in neuronal cells (Markmiller et al., 2018). Existing literature has found that SG formation attenuated cellular apoptosis by sequestering some apoptosis regulatory factors in the neuronal cell under stress (Arimoto-Matsuzaki et al., 2016), such as tumor necrosis factor (TNF) receptor-associated factor 2 (TRAF2), rho-associated coiled-coil containing kinase 1 (ROCK1), WD repeat-containing protein 62 (WDR62), and protein kinase C α (PKCα) (Kim et al., 2005; Kedersha et al., 2013). At present, no similar reports have been found to reveal the association between the anti-apoptotic effect of RBM3 and SGs formation, and the value deserves further study.

The leading cause of AIS injury is the reduction in cerebral blood flow and subsequent oxygen and glucose deprivation, and restoration (Su et al., 2017; Lin et al., 2018; Gregori-Pla et al., 2019). OGD/R (oxygen-glucose deprivation/reoxygenation) cell model is a recognized model that mimic the pathological features of ischemia-reperfusion injury *in vitro* (Luo et al., 2013; Wu et al., 2015; Gao et al., 2020). Therefore, we used OGD/R treated PC12 cells as the *in vitro* model of AIS.

In this study, we used the OGD/R model of rat primary cortical neurons and PC12 cells to mimic the pathological process of AIS and monitor the dynamic generation processes of SGs. Besides, the Co-IP (co-immunoprecipitation), RBM3 overexpression, and knockdown of RBM3 gene experiments were performed to further investigate the functions of RBM3 in SG formation and anti-apoptosis under ischemia-reperfusion injury condition.

## Materials and Methods

### Primary Cortical Neurons Culture

Six pregnant Sprague–Dawley rats at embryonic day 13 (E13) were purchased from the Animal Center of Guangzhou University of Chinese Medicine (Guangzhou, China). The experimental protocols were approved by the Animal Care and Use Committee of Guangzhou University of Chinese Medicine (approval reference number: 20200324002). Primary cortical neurons were dissociated from embryonic day 18 (E18) Sprague–Dawley rats, as described previously (Wang et al., 2014a,b). In brief, the cortical region was dissected from the brain of E18 embryos, cut into small pieces (1 mm cube), and digested with 0.25% trypsin under sterile condition. The digested cortices were dissociated by repeated passage through glass fire-polished Pasteur pipette and filtered through a 70 μm cell strainer (catalog no. 352350; BD Biosciences, Bedford, MA, United States). Approximate 2 × 10^5^ cells/cm^2^ were seeded onto poly-L-lysine (10 μg/ml)-coated plates then cultivated for 6 h in Dulbecco’s modified Eagle’s medium (DMEM, high glucose; catalog no. 11971025; Gibco, Thermo Fisher Scientific, Inc., United States) with 10% fetal bovine serum (FBS, catalog no. 16140071; Gibco, Thermo Fisher Scientific, Inc., United States). After 6 h of culture, neurons were incubated in neurobasal medium (catalog no. 21103049; Gibco, Thermo Fisher Scientific, Inc., United States) supplemented with 2% B27 (catalog no. 17504044; Invitrogen, Thermo Fisher Scientific, Inc.), 1% L-glutamine (catalog no. 35050061; Gibco, Thermo Fisher Scientific, Inc., United States), and 1% Pen Strep (catalog no. 15070063; Gibco, Thermo Fisher Scientific, Inc.). We renewed half of the medium every 2 days for 2 weeks. With using rabbit anti-MAP2 antibody (catalog no. 17490-1-AP; 1:500 dilution; Proteintech, Wuhan, China), Alexa Fluor488-conjugated goat anti-rabbit antibody (catalog no. ab150077; 1:1000 dilution; Abcam, Cambridge, United Kingdom), and DAPI staining (catalog no. D1306; 300 nM concentration; Invitrogen, Thermo Fisher Scientific, Inc., United States), we confirmed that ≥ 90% living cells were MAP2 positive neurons (Supplementary Material S1). Images were acquired using the confocal laser scanning microscope (LSM 800, Zeiss, Germany). Finally, the neurons were subjected to OGD/R treatment.

### Oxygen-Glucose Deprivation/Reperfusion (OGD/R) Injury

OGD/R is a well-established *in vitro* model to study the pathology of acute ischemic stroke (Grosenbaugh et al., 2018). The cell model of OGD/R injury was established with rat primary cortical neurons and PC12 cells (ATCC, Manassas, VA, United States) which underwent OGD/R injury as described previously (Polavarapu et al., 2007; Xie et al., 2012; Li et al., 2014). The grouping was as follows: control group; R-0 h group (OGD for 6 h and reperfusion for 0 h); R-6 h group (OGD for 6 h and reperfusion for 6 h); R-12 h group (OGD for 6 h and reperfusion for 12 h); R-18 h group (OGD for 6 h and reperfusion for 18 h); and R-24 h group (OGD for 6 h and reperfusion for 24 h). Briefly, after three-time washing with PBS, cortical neurons and PC12 cells were incubated in glucose-free RPMI 1640 medium (catalog no. 11879020; Gibco, Thermo Fisher Scientific, Inc., United States), and then placed in an anaerobic chamber (Coy Laboratory Products, Inc.) under an atmosphere of 95% N_2_/5% CO_2_ at 37°C for 6 h of OGD stimulation. At the end of OGD stimulation, the culture media of primary cortical neurons and PC12 cells were changed to neurobasal medium containing 2% B27 and RPMI 1640 medium (catalog no. 22400105; Gibco, Thermo Fisher Scientific, Inc., United States) with 10% fetal bovine serum under standard cell culture conditions (37°C, 5% CO_2_ and 95% air) for different time points reperfusion (0, 6, 12, 18, and 24 h), respectively. Each experiment was replicated three times, and data are presented as the mean ± SEM.

### Western Blotting Analysis

PC12 cells were seeded at a density of 0.5 × 10^6^ per well in a six-well plate. After 6 h of OGD/R treatment, PC12 cells were lysed with RIPA Lysis and Extraction Buffer (catalog no. 89901; Thermo Fisher Scientific, United States) for 20 min on ice. The supernatant containing total protein was harvested after centrifugation at 10,000 × *g* for 20 min at 4°C. Protein concentrations were determined using the bicinchoninic acid assay kit (catalog no. 23227; Thermo Fisher Scientific, United States). 30 μg of total protein was separated with 10% SDS-PAGE and transferred to 0.45 um PVDF membrane (catalog no. IPVH00010; Millipore, Germany). After blocking with 5% bovine serum albumin (BSA; catalog no. A1933; Sigma-Aldrich, United States) for 2 h at room temperature, the membrane was incubated with anti-RBM3 antibody (catalog no. 14363-1-AP; rabbit polyclonal; Proteintech, Wuhan, China; 1:1000 dilution), anti-BCL2 antibody (catalog no. 12789-1-AP; rabbit polyclonal; Proteintech, Wuhan, China; 1:1000 dilution), anti-Caspase 3 active antibody (catalog no. ab2302; rabbit polyclonal; Abcam, Cambridge, United Kingdom; 1:1000 dilution) and anti-GAPDH antibody (catalog no. ab245355; rabbit polyclonal; Abcam, Cambridge, United Kingdom; 1:5000 dilution; catalog no. 10494-1-AP; rabbit polyclonal; Proteintech, Wuhan, China; 1:5000 dilution) at 4°C overnight. After washing, the cells were incubated with secondary antibody (catalog no. ab6721; goat anti-rabbit IgG-HRP; Abcam, Cambridge, United Kingdom; 1:5000 dilution) for 1 h (RT). Enhanced chemiluminescence substrate (ECL substrate; catalog no. 32109; Pierce, Thermo Fisher Scientific, Inc., United States) was applied to visualize the protein bands. The gray density of protein bands was quantitatively analyzed by Image J (National Institutes of Health, Bethesda, MD, United States).

### Immunofluorescence Staining

Rat primary cortical neurons and PC12 cells were grown on glass coverslips in 24-well plates (2 × 10^5^ cells). After 6 h of OGD/R treatment, two kinds of cells were fixed with 4% paraformaldehyde for 20 min, permeabilized in 0.3% Triton X-100 solution for 10 min, and blocked with 10% goat serum (catalog no. 16210064; Gibco, Thermo Fisher Scientific, Inc., United States) for 1 h. The above steps were conducted at room temperature. The cells were then incubated with mixed primary antibodies: rabbit anti-TIA1 antibody (catalog no. ab140595; 1:250 dilution; Abcam, Cambridge, United Kingdom) and mouse anti-G3BP1 antibody (catalog no. ab56574; 1:250 dilution; Abcam, Cambridge, United Kingdom) at 4°C overnight. After washing, the cells were incubated with mixed secondary antibodies (catalog no. ab150077, Alexa Fluor488-conjugated goat anti-rabbit antibody, 1:1000 dilution; catalog no. ab150114, Alexa Fluor555-conjugated goat anti-mouse antibody,1:1000; Abcam, Cambridge, United Kingdom) in the dark for 1 h. DAPI staining (catalog no. D1306; 300 nM concentration; Invitrogen, Thermo Fisher Scientific, Inc., United States) for 10 min was used for nuclei labeling. Images were acquired using the confocal laser scanning microscope (LSM 800, Zeiss, Germany) at 400X magnification. Cells with at least two double-positive SGs (TIA1 and G3BP1-positive) were considered the SG-positive cells (Meyerowitz et al., 2011; Arimoto-Matsuzaki et al., 2016). SG-positive cells and DAPI-positive cells were counted using the Image-Pro Plus software (Media Cybernetics, United States). A minimum of 500 cells was counted across multiple fields of view. Data were expressed as a percentage of SG-positive cells against DAPI-positive cells:% positive cells = (number of double-positive cells/DAPI-positive cells × 100). Each experiment was replicated three times, and data are presented as the mean ± SEM.

### Flow Cytometry

The Apoptosis Detection kit (catalog no. 556547, BD Pharmingen, United States) was used to detect the apoptosis level in PC12 cells. In brief, PC12 cells (1 × 10^6^ cells) were digested with 0.25% EDTA-free trypsin (catalog no. 15050065; Gibco, Thermo Fisher Scientific, United States) and resuspended in 1 mL 1X binding buffer. Annexin V (5 μL) and propidium iodide (5 μL) were added to the cell suspension and incubated for 10 min. CytoFLEX flow cytometry (Beckman Coulter, Inc., United States) was performed to detect the cells’ fluorescence intensity. Each experiment was replicated three times, and data are presented as the mean ± SEM.

### Live/Dead Cell Staining

PC12 cells grown in 24-well microplates were treated with different time points reperfusion (0, 6, 12, 18, and 24 h) after 6 h of OGD stimulation, then stained with a LIVE/DEAD^TM^ Cell Imaging Kit (catalog no. R37601; Invitrogen, Thermo Fisher Scientific, United States). Images were captured with a Cytation5 image reader (BioTek, Winooski, VT, United States) at 100X magnification. A minimum of 500 cells was counted across multiple fields of view. Data were expressed as a percentage of viable cells: Percentage of live cells = (total Calcein AM positive cells)/(total Calcein AM positive cells + total PI-positive cells). Each experiment was replicated three times, and data are presented as the mean ± SEM.

### RBM3 Knockdown Experiment in PC12 Cells

Targeting sequences for CRISPR knockdown were designed using the CRISPR tool^1^. The knockdown of RBM3 in PC12 cells was generated with CRISPR-Cas9 gene-editing system by targeting the following sequence: 5′-GTAGC TGCGACCACGCCCATGGG-3′. Briefly, complementary oligonucleotides of RBM3 with *Bsa*I (New England Biolabs, Beijing, China) restriction sites for guide RNAs (gRNAs) were synthesized and inserted into the pGL3-U6-gRNA plasmid (a kind gift from Prof. Jiankui Zhou) and confirmed by sequencing. PC12 cells were seeded in 6-well plates (1 × 10^6^ cells/well). After 24 h of cultivation, PC12 cells were transfected with 10 μg pST1374-Cas9-ZF-NLS (a kind gift from Prof. Jiankui Zhou) and 10 μg pGL3-U6-gRNA (pGL3-U6-RBM3-gRNA) plasmid using Lipofectamine 3000 (catalog no. L3000015; 20 μL; Invitrogen; Thermo Fisher Scientific, Inc.). After 48 h of transfection, the puromycin-resistant cells were selected in RPMI-1640 complete medium with a concentration of 2 μg/mL of puromycin last for 7 days. The puromycin-resistant cells were transferred to a medium without puromycin and expanded. The efficient RBM3 knockdown was confirmed by western blotting assay. The primer sequences and plasmid maps are provided in Supplementary Material S2. Each experiment was replicated three times, and data are presented as the mean ± SEM.

### Overexpression of RBM3 in PC12 Cells

The RBM3 overexpression plasmids (pCDH-CMV-RBM3) applied in this study were designated and constructed (Shenzhen Huaan Ping Kang Bio Technology, Inc., Shenzhen, China). PC12 cells (0.5 × 10^6^ cells) were seeded in a six-well plate. When PC12 cells were 70–80% of confluence, they were transfected with 10 μg pCDH-CMV-RBM3 plasmids using Lipofectamine 3000 (catalog no. L3000015; 10 μL; Invitrogen; Thermo Fisher Scientific, Inc.) according to the manufacturer’s instructions. After 24 h of transfection, the puromycin-resistant cells were selected in RPMI-1640 complete medium with a concentration of 2 μg/mL of puromycin for 3 days. The puromycin-resistant cells were transferred to a medium without puromycin and expanded. Besides, the efficient RBM3 overexpression was confirmed by western blotting assay. The empty pCDH-CMV plasmid was used as the negative control (NC). Each experiment was replicated three times, and data are presented as the mean ± SEM.

### Co-immunoprecipitation (Co-IP)

Crosslink Magnetic IP/Co-IP Kit (catalog no. 88805; Pierce, Thermo Fisher Scientific, Inc., United States) was used to perform Co-IP analysis. According to the manufacturer’s protocol, 10 μg RBM3 antibody (RBM3 Rabbit pAb, Proteintech, Wuhan, China) was covalently cross-linked to 25 μL magnetic beads using disuccinimidyl suberate (DSS). The antibody-crosslinked beads were incubated with a total of 500 μg protein, which was extracted from PC12 cells and quantified by bicinchoninic acid assay kit (catalog no. 23227; Thermo Fisher Scientific, United States). After washing twice to remove non-bound material, the bound antigen of beads was yielded using a low-pH elution buffer and prepared for SDS-PAGE analysis.

### Statistical Analysis

The experimental data are presented as the mean ± SEM. SPSS software version 22.0 (SPSS, Inc. IBM Corporation) was used for statistical analyses. The normality of values was tested with the Shapiro–Wilk normality test. All figures were produced using GraphPad Prism (Version 6.0; GraphPad Software, Inc., La Jolla, CA, United States). Statistical differences between two groups of data were analyzed with a two-tailed unpaired *t*-test or non-parametric Mann–Whitney *U*-test. One-way ANOVA, two-way ANOVA, or Kruskal–Wallis non-parametric test was performed to investigate the difference between more than two groups followed by *post hoc* comparison (Tukey’s multiple comparisons test). A *P*-value of < 0.05 was considered statistically significant.

## Results

### SGs Dynamically Form at Different Time Points of Reperfusion Under OGD/R Injury Condition in Rat Primary Cortical Neurons

**FIGURE 1:**
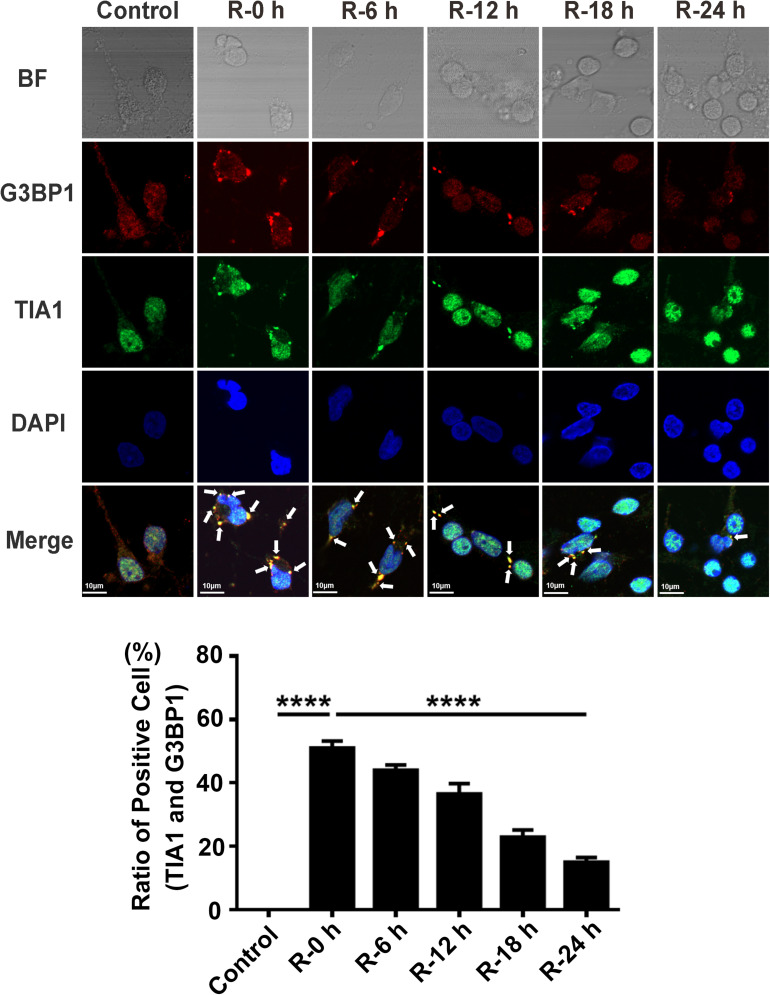
The dynamic generation of SGs at different time points of reperfusion under OGD/R condition in rat primary cortical neurons. SG formation of rat primary cortical neurons was detected with double-labeled immunofluorescence (TIA1 and G3BP1) at 0, 6, 12, 18, and 24 h reperfusion after 6 h OGD stimulation. ****, compared with R-0 h group; *P* < 0.0001. SGs were labeled with TIA1 (green) and G3BP1 (red), and the nucleus was labeled with DAPI (blue). Scale bars: 10 μm.

To study whether there is SGs formation under OGD/R injury condition, we used OGD/R (oxygen-glucose deprivation/reperfusion) stimulation in rat primary cortical neurons as an *in vitro* model of AIS. Double-labeled immunofluorescence (TIA1 and G3BP1) was used to observe SG formation at different time points of recovery (0, 6, 12, 18, and 24 h) after 6 h of OGD stimulation. The highest level of SG formation appeared at 0 h of reperfusion and then gradually decreased from 0 to 24 h of reperfusion after 6 h of OGD stimulation. The level of SG formation was significantly reduced from 50.97 to 14.97% (70.4%) in the R-24 h group compared with the R-0 h group (Figure 1; R-0 h vs. R-24 h; *P* < 0.0001). These data demonstrate that SGs dynamically generate under OGD/R injury in primary cortical neurons.

### SGs Dynamically Form at Different Time Points of Reperfusion Under OGD/R Injury Condition in PC12 Cells

**FIGURE 2:**
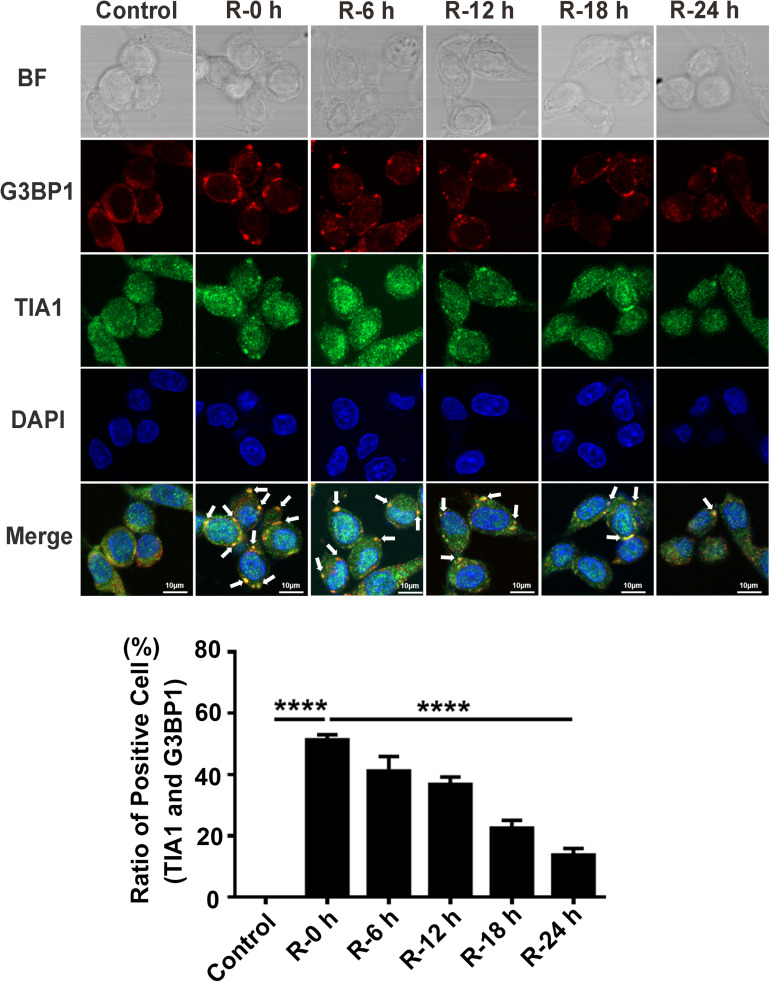
The dynamic generation of SGs at different time points of reperfusion under OGD/R condition in PC12 cells. SG formation in PC12 cells at 0, 6, 12, 18, and 24 h of reperfusion after 6 h of OGD stimulation was detected with double-labeled immunofluorescence (TIA1 and G3BP1). ****, compared with R-0 h group; *P* < 0.0001. SGs were labeled with TIA1 (green) and G3BP1 (red), and the nucleus was labeled with DAPI (blue). Scale bars: 10 μm.

We performed OGD/R (oxygen-glucose deprivation/recovery) stimulation in PC12 cells to verify the results observed in rat primary cortical neurons. Double-labeled immunofluorescence (TIA1 and G3BP1) was used to observe SG formation at different time points of recovery (0, 6, 12, 18, and 24 h) after 6 h of OGD stimulation. The results were similar to the outcomes of rat primary cortical neurons. The highest SG formation-level also appeared at 0 h of reperfusion and then gradually decreased from 0 to 24 h of reperfusion after 6 h of OGD stimulation. According to the results of the immunofluorescence, SG formation was significantly reduced from 51.30 to 13.77% (72.90%) in the R-24 h group compared with the R-0 h group in PC12 cells (Figure 2; R-0 vs. R-24 h; *P* < 0.0001). These data demonstrate that SGs dynamically generate under OGD/R injury in PC12 cells.

### Apoptosis Level at Different Time Points of Reperfusion Under OGD/R Injury Condition in PC12 Cells

**FIGURE 3:**
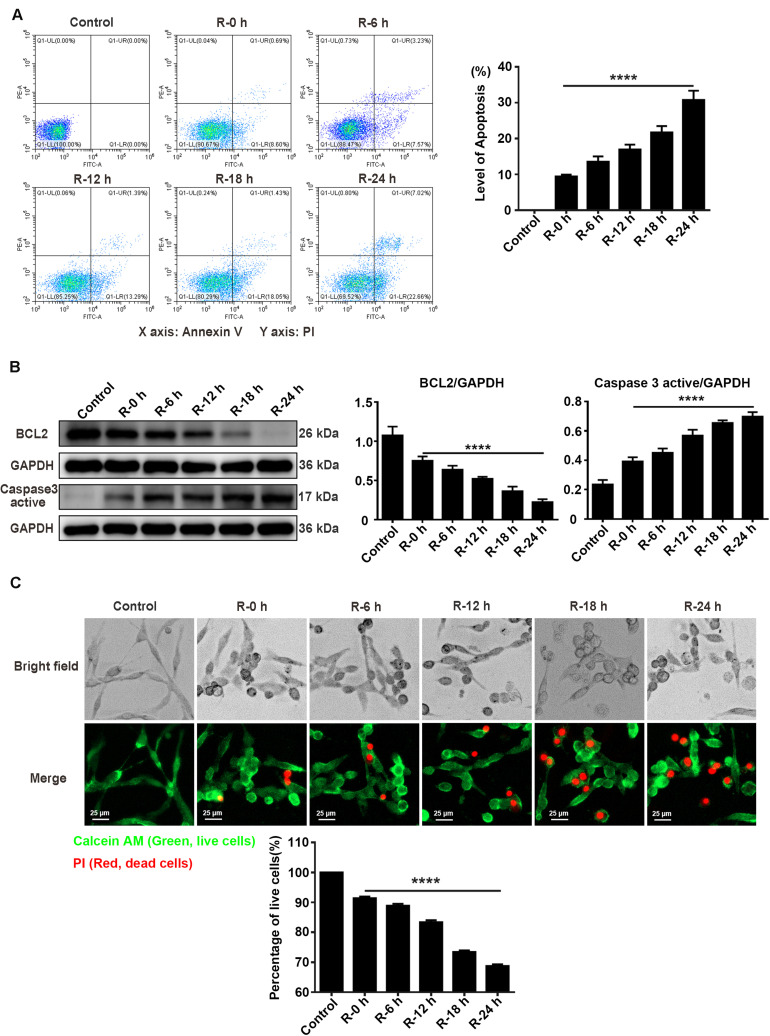
The apoptosis level and percentage of live cells at different time points of reperfusion under OGD/R injury condition in PC12 cells. **(A)** Apoptosis levels in PC12 cells at 0, 6, 12, 18, and 24 h of reperfusion after 6 h of OGD stimulation. Annexin V-FITC/PI double staining assay by the flow cytometry was used to detect the dynamic change of apoptosis levels. A total of 10,000 cells were counted for each flow cytometry analysis. ****, compared with R-0 h group; *P* < 0.0001. X-axis: Annexin V; Y-axis: PI. **(B)** BCL2 and Caspase 3 active protein expression at different time points of reperfusion. The western blotting assay was used to detect the protein expression BCL2 and Caspase 3 active at different time points (0, 6, 12, 18, and 24 h) of reperfusion after 6 h of OGD stimulation. ****, compared with R-0 h group; *P* < 0.0001. **(C)** The percentage of live cells at different time points of reperfusion under OGD/R injury condition. Live/Dead cell staining was used to measure the percentage of live cells at different time points of reperfusion. Cells were stained with Calcein AM (green; live cells) and PI (red; dead cells). ****, compared with R-0 h group; *P* < 0.0001. Scale bars: 25 μm.

To investigate the apoptosis level of PC12 cells treated with OGD/R stimulation, we used flow cytometry assays (annexin V/PI double staining), western blotting assays, and live/dead cell staining to observe the apoptosis level and percentage of live cells at different time points of reperfusion (0, 6, 12, 18, and 24 h) after 6 h of OGD stimulation. According to the flow cytometry assay results, apoptosis level was significantly decreased at R-0 h in PC12 cells compared with group R-24 h (Figure 3A; R-0 h vs. R-24 h: *P* < 0.0001). As presented in Figure 3B, BCL2 protein expression significantly decreased by 70.41% and the Caspase 3 active protein expression increased by 78.60% in R-24 h group compared with R-0 h group (Figure 3B; R-0 h vs. R-24 h, *P* < 0.0001; R-0 h vs. R-24 h, *P* < 0.0001). Live/dead cell staining showed that the percentage of live cells was significantly decreased from 91.39 to 68.74% in R-24 h group compared with R-0 h group (Figure 3C; R-0 h vs. R-24 h, *P* < 0.0001). These data demonstrated an inverse relationship between SG formation and apoptosis level *in vitro*, suggesting the protective effect of SGs under OGD/R injury in PC12 cells.

### Expression Levels of RBM3 in PC12 Cells Under OGD/R Stimulation

**FIGURE 4:**
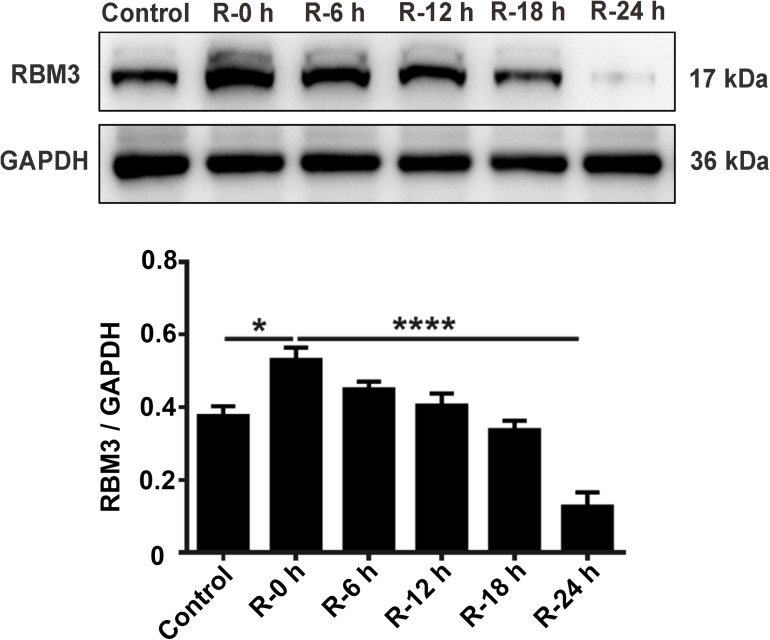
Expression levels of RBM3 in PC12 cells under OGD/R stimulation. Total protein was extracted from PC12 cells at different time points of reperfusion (0, 6, 12, 18, and 24 h) after 6 h of OGD stimulation. Western blot analysis was used to detect RBM3 expression. *, compared with R-0 h group; *P* < 0.05; ****, compared with R-0 h group; *P* < 0.0001.

To explore the protein expression levels of RBM3, we extracted the total protein from PC12 cells at different time points of reperfusion (0, 6, 12, 18, and 24 h) after 6 h of OGD stimulation and subjected to western blot analysis. Western blotting results showed that RBM3 expression increased at R-0 h, R-6 h, and R-12 h then decreased at R-18 h and R-24 h (Figure 4). RBM3 expression was significantly reduced by 75.1% in the R-24 h group than the R-0 h group (Figure 4; R-0 h vs. R-24 h: *P* < 0.0001). These data suggested that RBM3 expression increased in the early stage of reperfusion (R-0 h) after 6 h of OGD injury.

### RBM3 Interacts With G3BP1 in PC12 Cells Under OGD Stimulation

**FIGURE 5:**
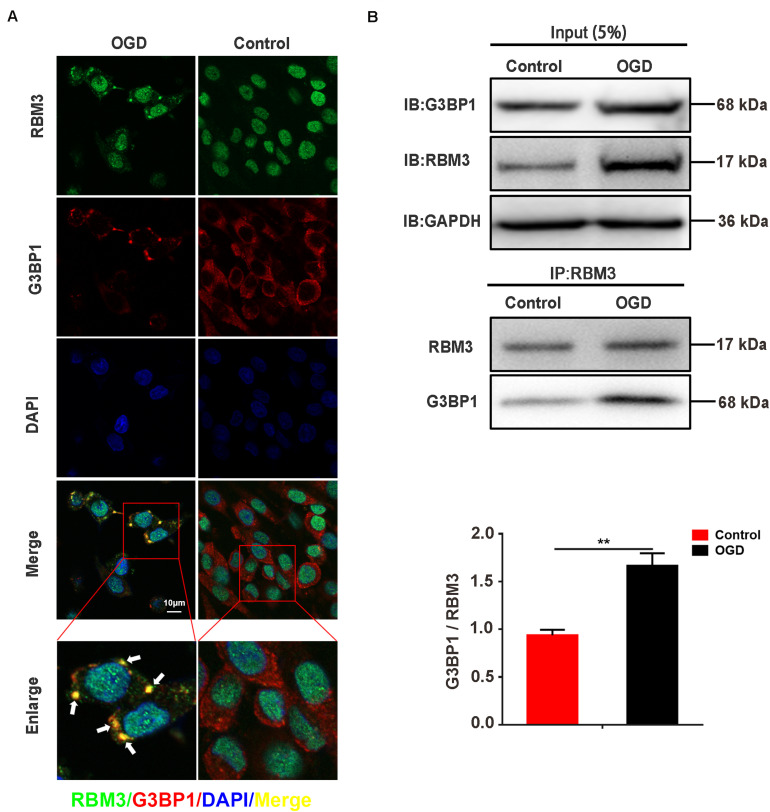
RBM3 interacts with G3BP1 in PC12 cells under OGD stimulation. **(A)** The colocalization of RBM3 with G3BP1. Representative merged images showing the colocalization of RBM3 with G3BP1 in PC12 cells under 6 h of OGD stimulation (Green represented RBM3; red represented G3BP1; yellow represented colocalization). **(B)** RBM3 interacted with G3BP1 in PC12 cells under OGD/R stimulation. The co-IP assay was used to detect the interaction between RBM3 and G3BP1 in PC12 cells after 6 h of OGD stimulation. **, compared with control group; *P* < 0.01.

To observe the intracellular localization of RBM3, we performed the immunofluorescence assay on PC12 cells with confocal laser microscopy. The results showed that RBM3 was colocalized with G3BP1 in PC12 cells under 6 h of OGD-stimulation (Figure 5A; yellow represented colocalization). To confirm the interaction between RBM3 and G3BP1, we performed co-immunoprecipitation (Co-IP) assay in PC12 cells under 6 h of OGD stimulation. The results suggested that the binding effect between RBM3 and G3BP1 significantly increased by 79.9% in the OGD group than the control group (Figure 5B; control vs. OGD: *P* < 0.01). These data suggested that RBM3 interacted with G3BP1 in PC12 cells under 6 h of OGD stimulation. The colocalization of RBM3 and G3BP1 in the cytoplasm may facilitate the SG formation.

### RBM3 Promotes SG Formation in PC12 Cells Under OGD Stimulation

**FIGURE 6:**
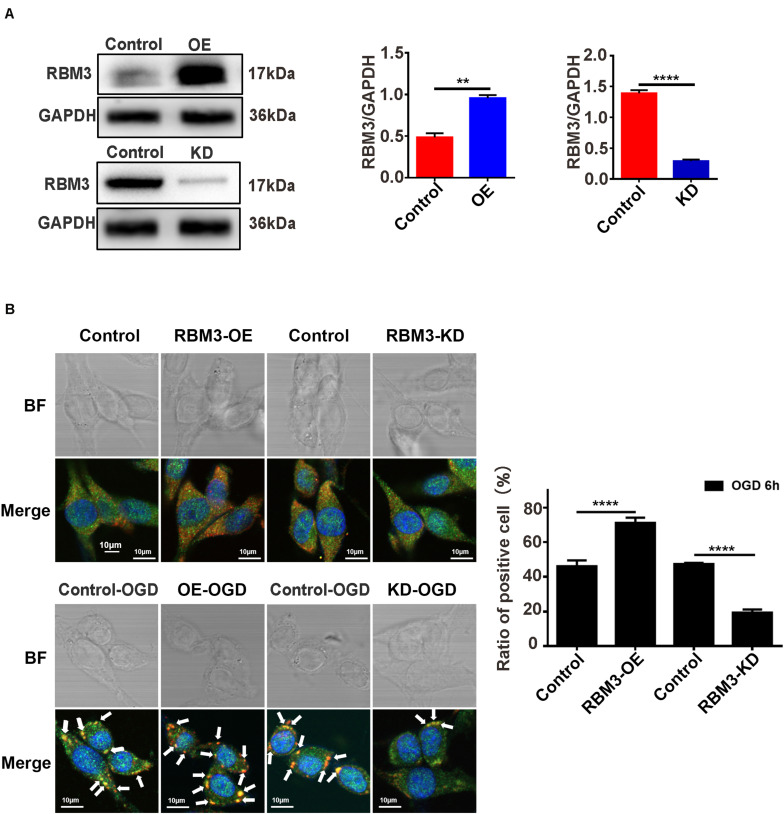
RBM3 promotes SG formation in PC12 cells under OGD stimulation. **(A)** The efficiency of RBM3 knockdown or overexpression in PC12 cells. The efficiencies of RBM3 knockdown and overexpression were detected by western blotting (****, control vs. KD: *P* < 0.0001; **, control vs. OE: *P* < 0.01). **(B)** SG formation in RBM3 knockdown or overexpression PC12 cells under OGD stimulation. SGs were detected with double-labeled immunofluorescence (TIA1 and G3BP1); the nucleus was labeled with DAPI (blue). Scale bars: 10 μm. ****, compared with control group; *P* < 0.0001.

To explore the role that RBM3 played in AIS injury, we performed the RBM3 knockdown and overexpression in PC12 cells. RBM3 knockdown and overexpression were generated by the CRISPR/Cas9 gene-editing system and transfection of overexpression plasmid in PC12 cells. Western blot assays validated the knockdown and overexpression efficiency of RBM3 in PC12 cells (Figure 6A, control vs. KD: *P* < 0.0001; control vs. OE: *P* < 0.01). Double-labeled immunofluorescence (TIA1 and G3BP1) assay was used to observe SG formation in RBM3 overexpressing cells and RBM3 knockdown cells under 6 h of OGD stimulation. The results of immunofluorescence demonstrated that the SG formation significantly increased from 46.17 to 71.16% (55.13%) in the RBM3-OE group compared with the control group (Figure 6B; control vs. RBM3-OE: *P* < 0.0001). Besides, the SG formation significantly reduced from 47.47 to 19.40% (59.12%) in the RBM3-KD group compared with the control group (Figure 6B, control vs. RBM3-KD: *P* < 0.0001). These data suggested that RBM3 plays a key role in promoting SG formation after 6 h of OGD stimulation.

### RBM3 Decreases Cell Apoptosis in PC12 Cells Under OGD Stimulation

**FIGURE 7:**
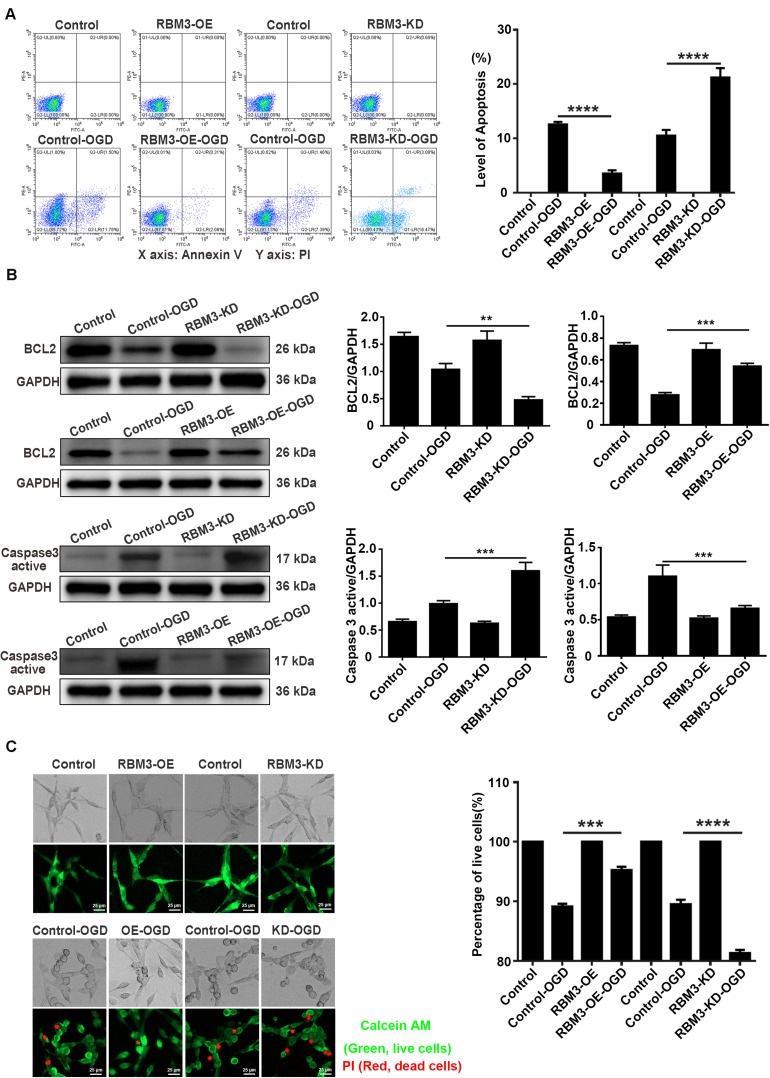
The apoptosis level and percentage of live cells in RBM3 overexpressing cells and RBM3 knockdown cells under 6 h of OGD/R injury. **(A)** Apoptosis levels of cells were detected by the annexin V-FITC/PI double staining assay. A total of 10,000 cells were counted for each flow cytometry analysis. ****, compared with control-OGD group; *P* < 0.0001. X-axis: Annexin V; Y-axis: PI. **(B)** BCL2 and Caspase 3 active protein expression in RBM3 overexpressing cells and RBM3 knockdown cells under 6 h of OGD/R injury. The western blotting assay was used to detect the protein expression BCL2 and Caspase 3 active. **, compared with control-OGD group; *P* < 0.01; ***, compared with control-OGD group; *P* < 0.001. **(C)** The percentage of live cells in RBM3 overexpressing cells and RBM3 knockdown cells under 6 h of OGD/R injury. Live/Dead cell staining was used to measure the percentage of live cells. Cells were stained with Calcein AM (green; live cells) and PI (red; dead cells). ***, compared with control-OGD group; *P* < 0.001; ****, compared with control-OGD group; *P* < 0.0001. Scale bars: 25 μm.

Flow cytometry assay (annexin V/PI double staining), western blotting assays, and live/dead cell staining were used to observe the apoptosis level and percentage of live cells in RBM3 overexpressing cells and RBM3 knockdown cells under 6 h of OGD stimulation. As shown in Figure 7A, the apoptosis level of cells was significantly reduced from 12.58 to 3.52% (71.6%) in the RBM3-OE-OGD group compared with the control-OGD group (Figure 7A; control-OGD vs. RBM3-OE-OGD; *P* < 0.0001). In addition, the apoptosis level was significantly increased from 10.51 to 21.23% (103.1%) in RBM3-KD-OGD group compared with the control-OGD group (Figure 7A; control-OGD vs. RBM3-KD-OGD; *P* < 0.0001). According to the results of western blotting assays, BCL2 protein expression significantly decreased by 53.92% and the Caspase 3 active protein expression increased by 61.38% in the RBM3-KD-OGD group compared with the control-OGD group (Figure 3B; control-OGD vs. RBM3-KD-OGD, *P* < 0.01; control-OGD vs. RBM3-KD-OGD, *P* < 0.001). In addition, BCL2 protein expression significantly increased by 94.99%, and the Caspase 3 active protein expression decreased by 39.76% in the RBM3-OE-OGD group compared with the control-OGD group (Figure 7B; control-OGD vs. RBM3-OE-OGD, *P* < 0.001; control-OGD vs. RBM3-OE-OGD, *P* < 0.001). As presented in Figure 7C, compared with the control-OGD group, the percentage of live cells was significantly increased from 89.14 to 95.25% (6.86%) in RBM3-OE-OGD group, and decreased from 89.53 to 81.31% (9.16%) in RBM3-KD-OGD group (Figure 7C; control-OGD vs. RBM3-OE-OGD, *P* < 0.001; control-OGD vs. RBM3-KD-OGD, *P* < 0.0001). These data demonstrated that RBM3 reduce the apoptosis level of PC12 cells under 6 h of OGD stimulation.

## Discussion

Due to the same neurological function as neuroendocrine cells, PC12 cells are widely used in neuropathological studies (Tabakman et al., 2005; Kikuchi et al., 2009; Guo et al., 2014; Gao et al., 2020). Here, we identified an essential role of RNA binding protein motif 3 (RBM3) in promoting SG formation via interacting with G3BP1 (GTPase-activating protein-binding protein 1) in OGD/R (oxygen-glucose deprivation/reperfusion) treated PC12 cells. RBM3-mediated SG formation provides further insight into the pathogenesis of ischemia-reperfusion injury. Our main findings can be outlined as follows: (i) SGs were generated under the early stage of ischemia-reperfusion condition; (ii) RBM3 binding with G3BP1 promoted SGs formation in the early stage of the ischemia-reperfusion disease.

One of the aims of this study was to evaluate the SGs formation under ischemia-reperfusion condition. Emerging evidence suggests that SGs formation and ischemia-reperfusion onset may occur concurrently in principle because of the similarity in the two events (Lee and Seydoux, 2019; Wolozin and Ivanov, 2019). Our previous research demonstrates that the marker of stress granules [T-cell intracellular antigen 1 (TIA1)] is upregulated at the early time-points of blood reperfusion after 2 h of cerebral ischemia, accompanying a significant decrease of apoptosis level in the ischemic cortex (Si et al., 2019). This finding hint that SGs formation may occur in the early state of AIS, but the direct evidence is still lacking. In the present study, as direct evidence, the highest level of SGs generation was observed with double-labeled immunofluorescence (G3BP1 and TIA1) in OGD/R treated PC12 cells after 6 h of OGD stimulation. And then, the SGs formation decreases gradually from 6 to 24 h after reperfusion. This finding confirms the SGs formation under ischemia-reperfusion condition can stimulate and provides a novel explanation for the host defense response to ischemia-reperfusion injury. Numerous literature reports have demonstrated that ischemic stroke damage is irreversible (Sun et al., 2013; Feng et al., 2014; Zhong et al., 2019). Therefore, it is meaningful to study the self-defense response mechanism to the ischemia-reperfusion environment and enhance cellular self-protection in ischemia-reperfusion conditions. It should be noted that the regulation of SGs formation could be a promising intervention to increase cellular self-defense and decrease the apoptosis level in ischemia-reperfusion injury.

Another primary aim of this study focused on the association between RBM3 and SGs formation under ischemia-reperfusion condition. First, our experimental data of RBM3 gene knockdown and overexpression suggested that RBM3 promoted SGs and reduced the apoptosis level in OGD/R treated PC12 cells. Consistent with the previous study, RBM3 was identified in the SGs and increased the production of SGs (Markmiller et al., 2018). By contrast, the percentage of SGs-positive cells was markedly increased in the RBM3 overexpression group and reduced in the RBM3 knockdown group compared with each control group. As a newly positive regulator of SGs formation, the identification of RBM3 may have high relevance to improving the understanding of the process of SGs assembly. The promoting effect of RBM3 in SGs formation provides a novel insight into understanding the protective effect of RBM3 on brain injury (Zhuang et al., 2017; Jackson et al., 2019; Yang et al., 2019; Zhu et al., 2019). Second, Co-IP experiments revealed that the interaction between RBM3 and G3BP1 significantly increase under OGD/R condition. This result suggests that induced RBPs – RBPs interactions under ischemia-reperfusion condition promote the SG formation in PC12 cells. Furthermore, this finding is supported by the previous findings of proteomic analysis of stress granule cores, which reveals a dense network of RBPs interactions, including RBM3 against oxidative stress induced by sodium arsenite (Jain et al., 2016). The association between RBM3 and G3BP1 could potentially be a critical factor in the SGs formation. As the core component of SGs, the slight conformational changes of G3BP1 triggering by the binding of RBM3 may facilitate the formation of the core of SGs and finally promoted the SGs generation under ischemia-reperfusion condition.

In summary, the central question of the present study focuses on the relationship between RBM3 and the SG formation under acute ischemic/reperfusion (I/R) conditions. Our findings indicate that RBM3 exerts a protective function against ischemia/reperfusion injury by promoting SGs formation via binding with G3BP1, thus strengthening the anti-apoptosis ability of PC12 cells under OGD/R stress. The significance of the current study is that it further our understanding of the underlying mechanisms of RBM3 in SG formation in rat primary cortical neurons and PC12 under OGD/R stress. The finding of RBM3 in promoting SGs formation may provide a promising target for alleviating ischemia-reperfusion injury.

## Data Availability Statement

The datasets generated for this study are available on request to the corresponding author.

## Ethics Statement

The animal study was reviewed and approved by the Ethical Committee of Guangzhou University of Chinese Medicine.

## Author Contributions

WS performed the experiments and wrote the manuscript. ZL performed CRISPR/Cas9 gene-editing assays and Co-IP assays. ZH performed the immunofluorescence assays and western blotting assays. SY performed the flow cytometry analysis. XL performed the Live/Dead cell staining. YL performed the immunofluorescence assays. WK provided the reagents and materials. MZ and DC designed the experiments and gave experimental technical guidance. All authors have read and approved the final manuscript.

## Conflict of Interest

The authors declare that the research was conducted in the absence of any commercial or financial relationships that could be construed as a potential conflict of interest.

## Footnotes

1 http://crispr.mit.edu
