# Supplementary material for: RNA Binding Protein Motif 3 Inhibits Oxygen-Glucose Deprivation/Reoxygenation-Induced Apoptosis Through Promoting Stress Granules Formation in PC12 Cells and Rat Primary Cortical Neurons
Source: Front Cell Neurosci. 2020 Sep 2;14:559384. doi: 10.3389/fncel.2020.559384 (PMC7492797; doi:10.3389/fncel.2020.559384)
Supplement: Supplementary file 1 [file Data_Sheet_1.PDF]

Supplementary Material S1

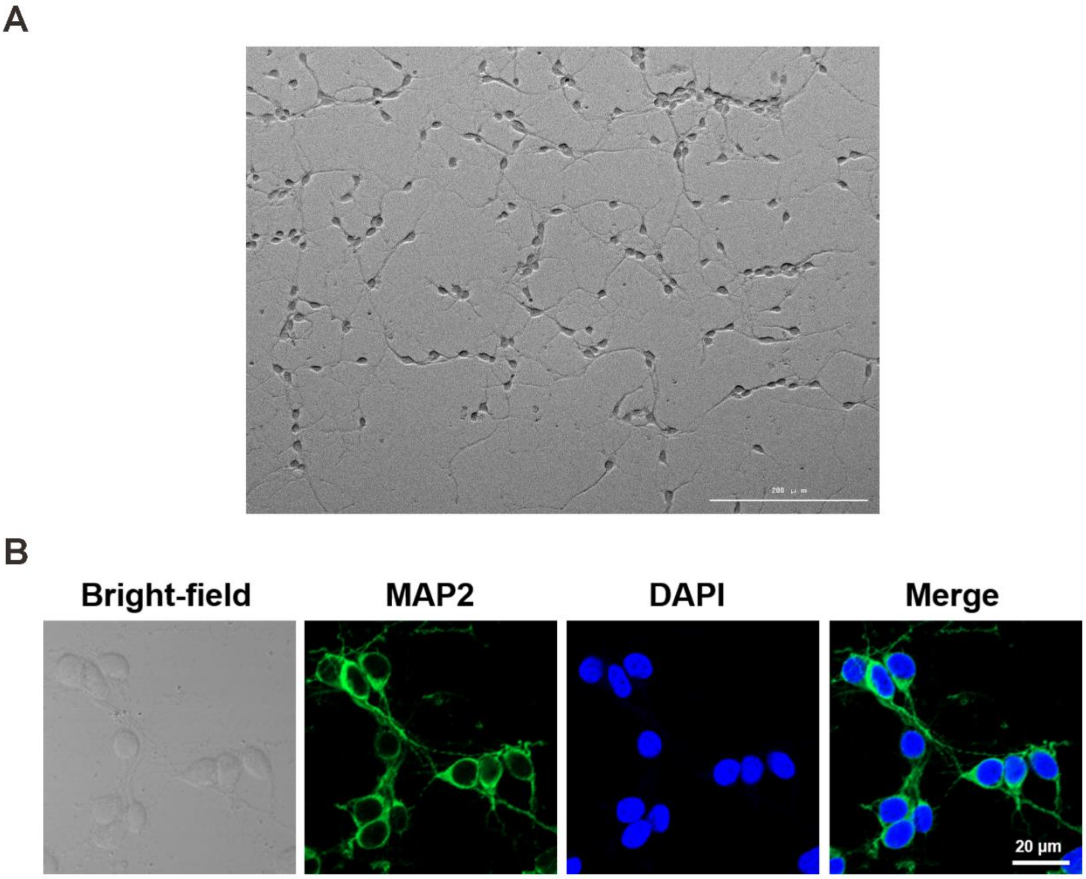

Figure S1 Detection of the purity of rat cortical neurons. (A) Bright-field pictures of rat cortical neurons. The image was captured with Cytation5 image reader (BioTek, Winooski, VT, USA) at 100X magnification. (B) MAP2 staining of rat cortical neurons. The neurons were stained with the dendritic protein MAP2 (catalog no. 17490-1-AP; rabbit polyclonal; Proteintech, Wuhan, China; 1:1000 dilution). MAP2 staining (green) was used to visualize neurons and DAPI staining (blue) to visualize nuclei. Images were acquired using the confocal laser scanning microscope (LSM 800, Zeiss, Germany) at 200X magnification. The staining confirmed at least 90% purity of rat cortical neurons.
