# Supplementary material for: RNA Binding Protein Motif 3 Inhibits Oxygen-Glucose Deprivation/Reoxygenation-Induced Apoptosis Through Promoting Stress Granules Formation in PC12 Cells and Rat Primary Cortical Neurons
Source: Front Cell Neurosci. 2020 Sep 2;14:559384. doi: 10.3389/fncel.2020.559384 (PMC7492797; doi:10.3389/fncel.2020.559384)
Supplement: Supplementary file 2 [file Data_Sheet_2.PDF]

Supplementary Material S2

	Name	Forward sequence (5′-3′)	Reverse sequence (5′-3′)
1	RBM3-sgRNA	accgtagctgcgaccacgcccat	aaacatgggcgtggtcgcagcta
2	RBM3-sg-PCR	ctccttcccatacctagtccct	cacctgcagaaaaacattgaag

Table S2 Primers used for RBM3 Knockdown in PC12 cells. RBM3-sgRNA was the complementary oligonucleotides of the target sequences of RBM3 with BsaI restriction sites (small guide RNAs, sgRNAs) and inserted into the pGL3-U6-gRNA plasmid. RBM3-sg-PCR was used for the amplification of target sequences and then sent for DNA sequencing analysis.

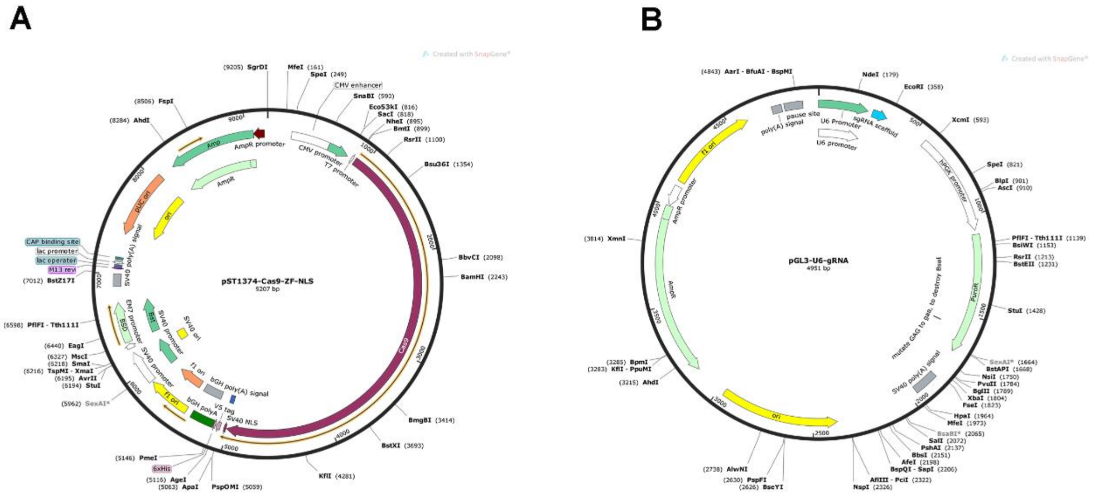

Figure S2 The plasmid maps used for RBM3 Knockdown. (A) The plasmid maps of pST1374-Cas9-ZF-NLS. (B) The plasmid maps of pGL3-U6-gRNA.
