# Supplementary material for: RNA Binding Protein Motif 3 Inhibits Oxygen-Glucose Deprivation/Reoxygenation-Induced Apoptosis Through Promoting Stress Granules Formation in PC12 Cells and Rat Primary Cortical Neurons
Source: Front Cell Neurosci. 2020 Sep 2;14:559384. doi: 10.3389/fncel.2020.559384 (PMC7492797; doi:10.3389/fncel.2020.559384)

Supplementary Material S3 (The bands of western blotting)

BCL2 (Figure 3B)

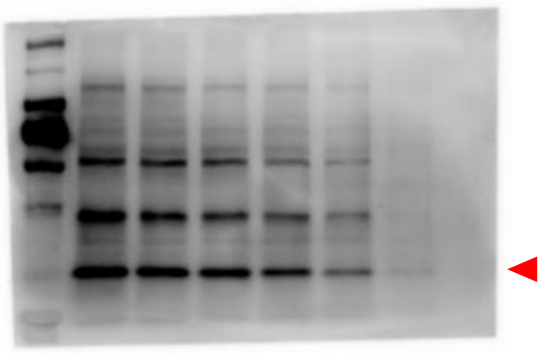

BCL2-GAPDH (Figure 3B)

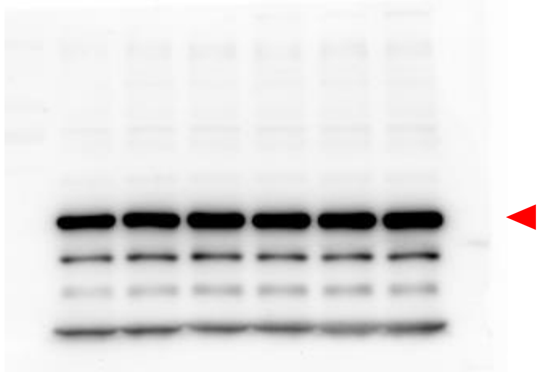

Caspase 3 active (Figure 3B)

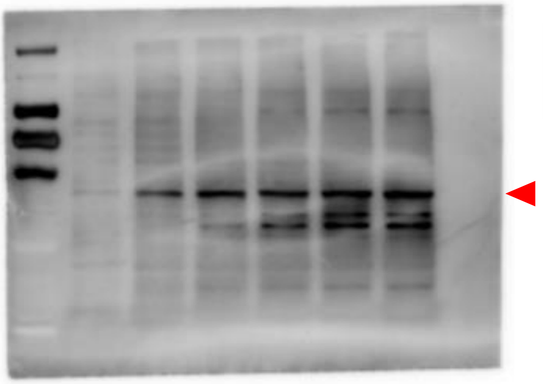

Caspase 3 active-GAPDH (Figure 3B)

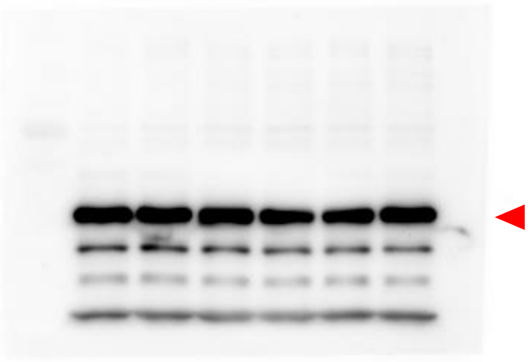

RBM3 (Figure 4)

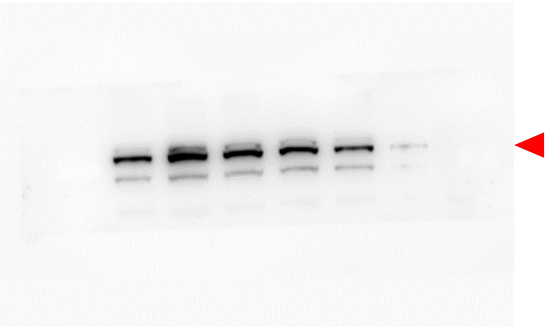

RBM3-GAPDH (Figure 4)

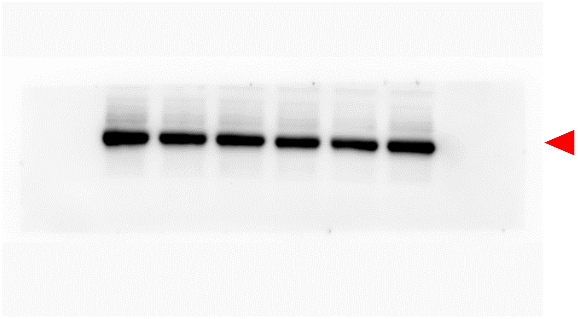

RBM3-IB (Figure 5B)

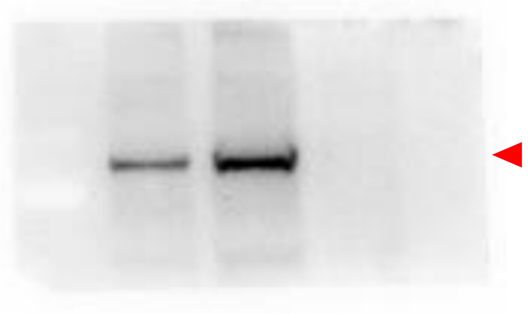

G3BP1-IB (Figure 5B)

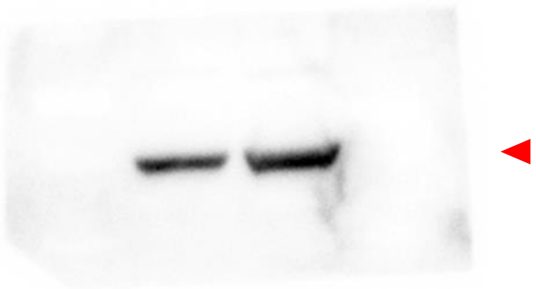

GAPDH-IB (Figure 5B)

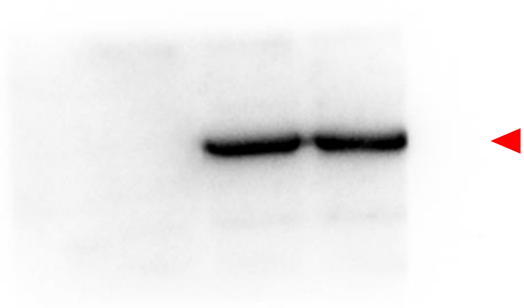

G3BP1-IP (Figure 5B)

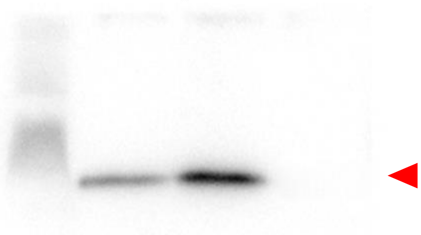

RBM3-IP (Figure 5B)

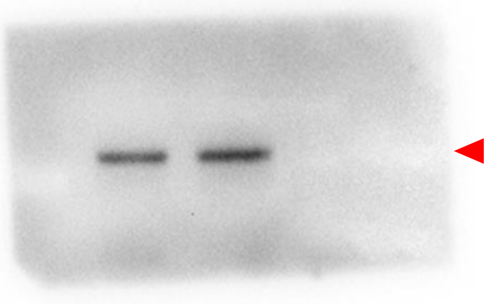

RBM3-KD (Figure 6A)

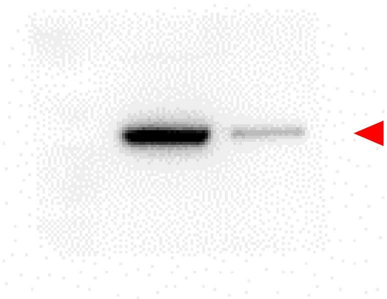

RBM3-OE (Figure 6A)

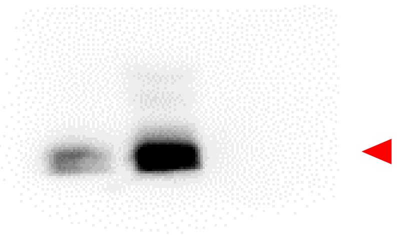

GAPDH (Figure 6A)

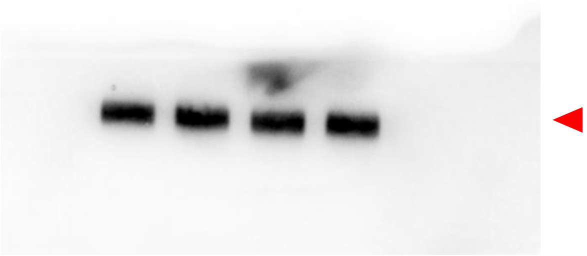

BCL2-OE (Figure 7B)

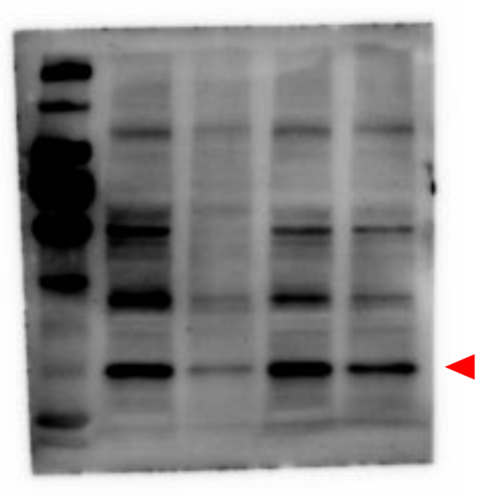

BCL2-OE-GAPDH (Figure 7B)

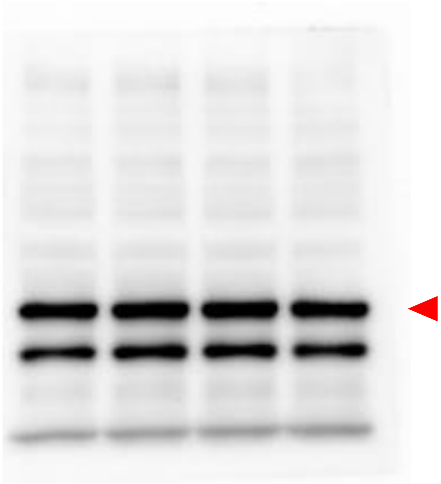

BCL2-KD (Figure 7B)

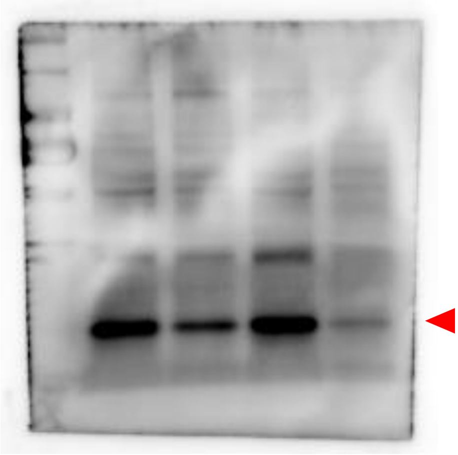

BCL2-KD-GAPDH (Figure 7B)

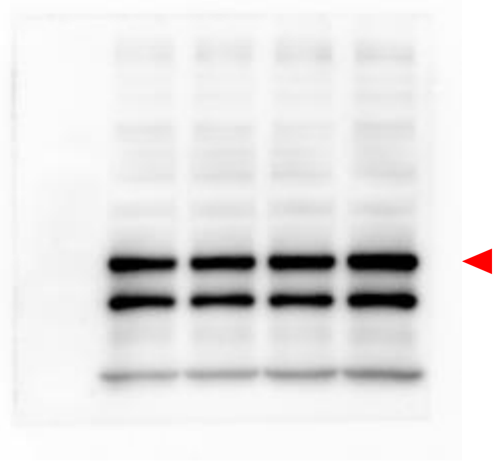

Caspase 3 active-KD (Figure 7B)

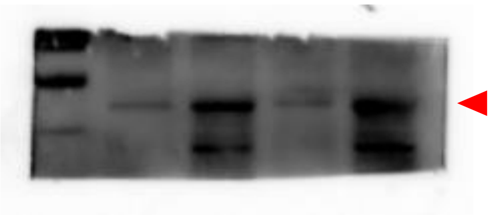

Caspase 3 active-KD-GAPDH (Figure 7B)

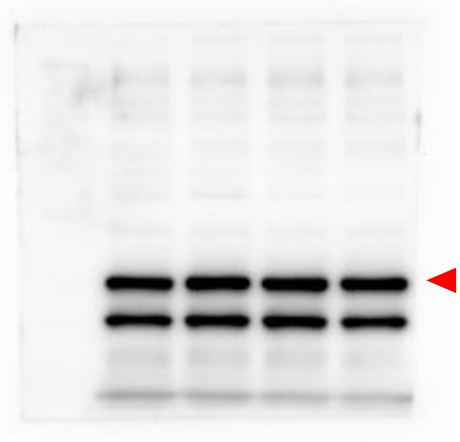

Caspase 3 active-OE(Figure 7B)

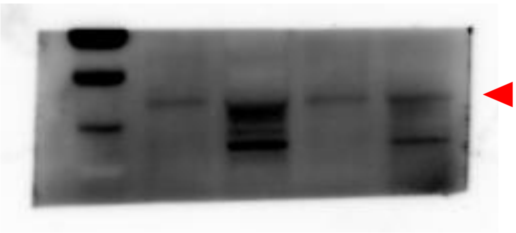

Caspase 3 active-OE-GAPDH (Figure 7B)

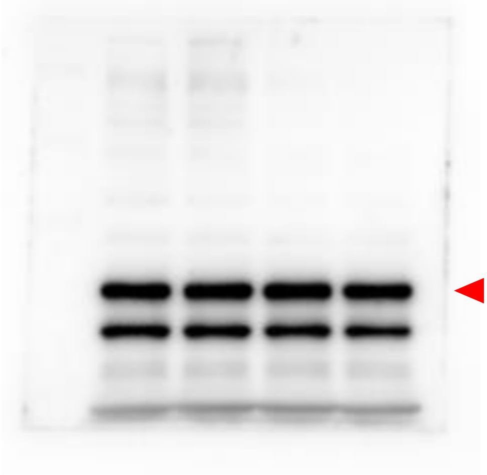

Supplement: Supplementary file 3 [file Data_Sheet_3.PDF]
