# Supplementary material for: RNA Binding Protein Motif 3 Inhibits Oxygen-Glucose Deprivation/Reoxygenation-Induced Apoptosis Through Promoting Stress Granules Formation in PC12 Cells and Rat Primary Cortical Neurons
Source: Front Cell Neurosci. 2020 Sep 2;14:559384. doi: 10.3389/fncel.2020.559384 (PMC7492797; doi:10.3389/fncel.2020.559384)
Supplement: Supplementary file 4 [file Data_Sheet_4.PDF]

Supplementary Material S4

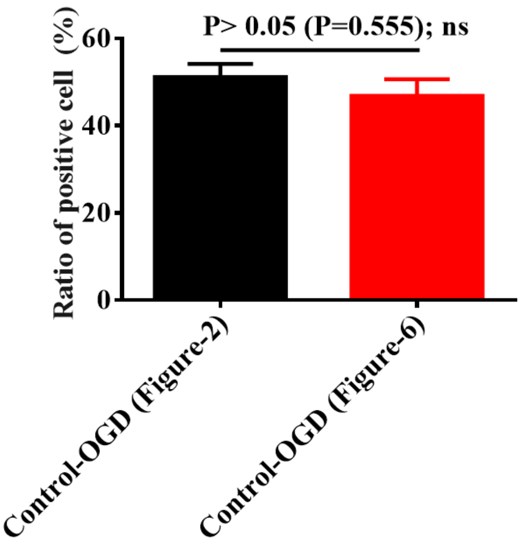

We repeated the same immunofluorescence staining of SGs in Figure 2 and Figure 6 and then analyzed the data of control-OGD groups together. However, the difference between the two groups was not statistically significant (Figure 2 vs. Figure 6, P=0.555).
