# Supplementary material for: RNA Binding Protein Motif 3 Inhibits Oxygen-Glucose Deprivation/Reoxygenation-Induced Apoptosis Through Promoting Stress Granules Formation in PC12 Cells and Rat Primary Cortical Neurons
Source: Front Cell Neurosci. 2020 Sep 2;14:559384. doi: 10.3389/fncel.2020.559384 (PMC7492797; doi:10.3389/fncel.2020.559384)
Supplement: Supplementary file 5 [file Data_Sheet_5.PDF]

Supplementary Material S5

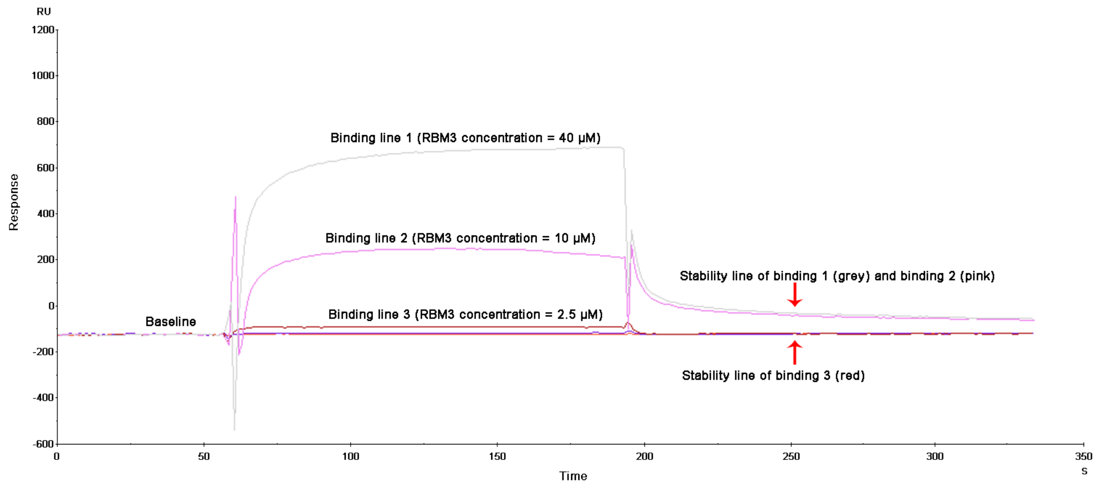

Surface plasmon resonance spectroscopy (SPR)

G3BP1 fusion protein (catalog no. Ag3728; Proteintech, Wuhan, China) was immobilized on a CM7 chip, and RBM3 fusion protein (catalog no. Ag28329; Proteintech, Wuhan, China) as analyte was injected over the chip. To analyze the binding ability, three concentrations of the RBM3 fusion protein (2.5 µM, 10 µM, and 40 µM) of were diluted in HBS-EP buffer (0.01 M HEPES, pH 7.4) and infused onto the sensor chip at a flow rate of 30 µL/min for 120 s. The response unit (RU) was recorded by Biacore (Biacore X-100, GE Healthcare Inc.). After the analyte infusion was stopped, the HBS-EP buffer was poured over the chip for 120 s at a flow rate of 30 µL/min. In comparison with the baseline, there is an increase of RU (59.0) in stability stage (10 µM and 40 µM concentrations of RBM3) after 120 s of chip washing. This result shows that RBM3 binds directly to the G3BP1.
